# Wearable sensors for monitoring caregivers of people with dementia: a scoping review

**DOI:** 10.1007/s41999-024-01113-8

**Published:** 2024-12-03

**Authors:** Francesco Palmese, Ylenia Druda, Vittoria Benintende, Domenico Fuda, Marcello Sicbaldi, Paola Di Florio, Abdul Haleem Butt, Giorgio Bedogni, Lorenzo Chiari, Alessandro Silvani, Marco Domenicali

**Affiliations:** 1 Department of Medical and Surgical Sciences, University of Bologna, Bologna, Italy; 2 Aging Research Center, Department of Neurobiology, Care Sciences and Society, Karolinska Institutet and Stockholm University, Stockholm, Sweden; 3 Department of Primary Health Care, Internal Medicine Unit Addressed to Frailty and Aging, “S. Maria Delle Croci” Hospital, AUSL Romagna, Ravenna, Italy; 4 Department of Electrical, Electronic and Information Engineering, University of Bologna, Bologna, Italy; 5 Department of Biomedical and Neuromotor Science, University of Bologna, Ravenna Campus, Ravenna, Bologna, Italy

**Keywords:** Caregivers, Dementia, Wearable sensors, Sleep analysis, Rest-activity, Physical activity

## Abstract

**Purpose:**

Caregivers of people with dementia often face a demanding care burden that may lead to adverse physical and psychological outcomes. Wearable sensors are becoming a valuable tool for preventive care. We aimed to provide the first systematic map of the published evidence on the use of wearable sensors to monitor caregivers of people with dementia.

**Methods:**

We developed a scoping review protocol following the PRISMA-ScR guidelines. The searches were conducted on PubMed, Scopus, Web of Science, PsycInfo, and IEEE.

**Results:**

We identified 1394 articles and selected 37 reports. Sleep measures were the outcome most frequently evaluated (28/37). Among these reports, eight assessed the effectiveness of different interventions targeting both caregivers and care-receivers and found no improvement in caregivers’ sleep; three compared the sleep of caregivers of people with dementia with controls, with conflicting results; five focused on depressive symptoms; and four associated these symptoms with shorter sleep duration and greater sleep fragmentation and sleep latency. A single device was used in 33 reports and sensors were most commonly placed at the wrist (33/37). Most studies monitored caregivers for 1 or more weeks (27/37).

**Conclusion:**

Wearable sensors were mainly used to monitor sleep in informal caregivers of people with dementia based on wrist accelerometry for 1 or more weeks, with conflicting results. There is a need for targeted studies with adequate control groups to identify factors associated with sleep alterations in informal and formal caregivers of people with dementia, leveraging multi-sensor setups with the inclusion of autonomic nervous system metrics.

**Supplementary Information:**

The online version contains supplementary material available at 10.1007/s41999-024-01113-8.

## Key summary points

**Aim:**

This scoping review aims to provide a systematic map of the published evidence regarding the use of wearable sensors in caregivers of people with dementia.

**Findings:**

Wearable sensors were mainly used to monitor sleep and physical activity in informal caregivers of people with dementia based on wrist accelerometry for one or more weeks, with conflicting results.

**Message:**

For caregivers of people with dementia, the potential applications of wearable sensors remain incompletely explored, with significant gaps in our understanding due to limitations of the available evidence.

**Supplementary Information:**

The online version contains supplementary material available at 10.1007/s41999-024-01113-8.

## Introduction

### Background

Dementia is a serious health issue that affects about 50 million adults worldwide and is expected to increase sharply by 2050 because of population aging [1]. Many people with dementia live in the community and need high levels of assistance for all activities of daily living. Such assistance is mainly provided by formal or informal caregivers [2]. The caregivers of people with dementia often face a demanding care burden that may lead to adverse physical and psychological outcomes [3].

Technological advancements over the past decade have resulted in the availability of wearable technology that can provide both assistance and monitoring to individuals living with chronic diseases, in accordance with the “4P medical model,” which stipulates that medicine should be preventive, predictive, personalized, and participative [4–6]. Wearable devices can track movement and gather information about people’s lifestyles and habits such as their mobility levels [6] or real-world walking speed [7], using miniature motion sensors like accelerometers or gyroscopes. Thus, wearable sensors could give healthcare providers valuable indicators regarding care receivers to inform care strategies and support the care process [8]. By the same token, wearable sensors may be employed to monitor the health status and well-being also of caregivers of people with chronic diseases, tracking the toll imposed by the burden of care and potentially allowing for early identification of burnout and other adverse health consequences.

The evidence on the use of wearable sensors is available in people with dementia, centered on sleep quality analysis, behavioral and psychological disorders, and physical activity [9–11]. Even though it is known that the health and well-being outcomes of people with dementia are connected with those of their caregivers, wearable sensor use, specifically in the caregivers of people with dementia, has received less attention [12–14]. Therefore, this scoping review aims to provide a systematic map of the published evidence regarding the use of wearable sensors in caregivers of people with dementia.

### Aims

The review was designed to answer the following questions:

- What is the available evidence regarding the use of wearable sensors by caregivers of people with dementia?
- Which sensor technologies (e.g., inertial measurement units, photoplethysmography), sensor locations (e.g., wrist, belt), and sensed variables (e.g., acceleration, heart rate) have been included in published reports on caregivers of people with dementia?
- What is the feasibility, usability, and acceptability of wearable sensors as assessed by caregivers of people with dementia?
- How does information on caregivers of people with dementia, measured with wearable sensors, affect caregiver quality of life, anxiety, depression, burden of care, stress, or any other outcomes related to caregiver health and well-being?
- What evidence is available about how information obtained with wearable sensors on caregivers of people with dementia impacts their care receivers?

## Methodology

The study protocol for this review was developed according to the PRISMA-ScR guidelines [15]. Although designed for systematic reviews, the PRISMA-P guidelines were also applied to this scoping review [16]. The study protocol was deposited on the Open Science Foundation website (https://doi.org/10.17605/OSF.IO/M72E4).

### Information sources

In April 2024, systematic electronic searches were conducted in the following databases:

- MEDLINE and PubMed Central (PMC), searched through PubMed;
- SCOPUS;
- Web of Science, searched through Clarivate Analytics;
- PsycInfo, searched through EBSCOHost;
- IEEE.

The selection of search terms and the construction of search queries were accomplished through collaborative efforts between members of the working group and an experienced librarian. Supplementary Table 1 (S1) provides the search strategy for each database.

### Eligibility criteria

The review included papers focusing on the use of wearable sensors by caregivers of people with dementia. The wearable sensors are devices that measure the physical signals of the human body for a given amount of time [17]. Both formal and informal caregivers and all stages and types of dementia were considered. We checked papers written in English, German, French, and Italian.

Conference abstracts, conference proceedings (except those published in the IEEE database), non-peer-reviewed articles, dissertations, theses, books, research protocols, letters, and editorials were excluded.

### Selection of sources of evidence and data charting process

After removing duplicates, each paper was screened independently by two reviewers (VB and DF) using Rayyan software [18]. The papers judged to meet the screening inclusion criteria by at least one reviewer were evaluated by two independent reviewers (FP and YD) based on their full text. The selection of the papers to be included in the review was made by consensus between the two reviewers involved in the selection process and after discussion with the entire research team.

## Results

### Search results

**Fig. 1:**
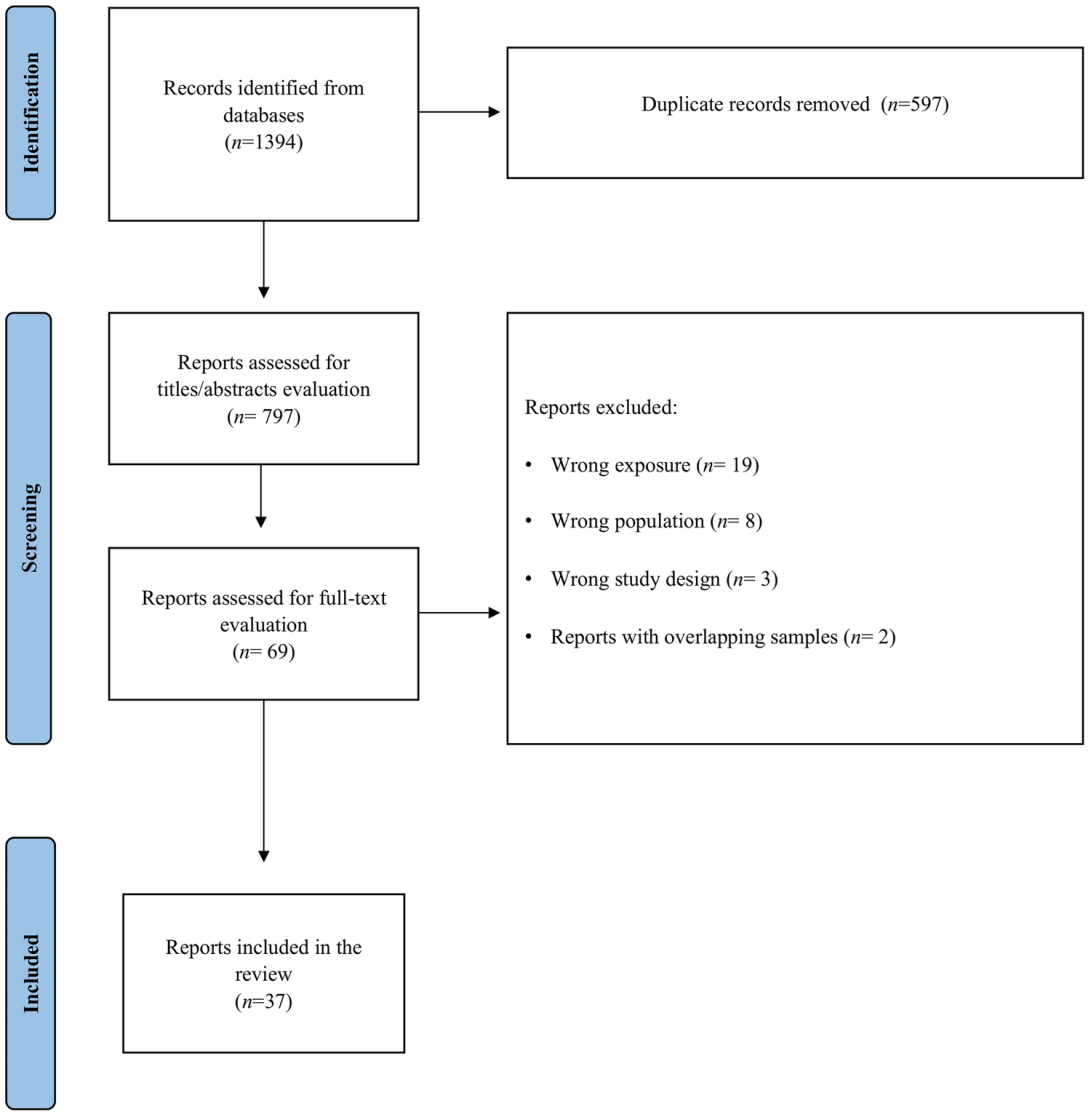
PRISMA flow diagram of the study selection process

The study selection process is summarized in Fig. 1. We identified 1394 papers from the online databases; we short-listed 69 papers after screening and selected 37 papers after full-text evaluation.

### Characteristics of the included studies

The characteristics of the studies selected for inclusion are described in Table 1.

**Table 1:** Main characteristics of the selected studies on wearable sensors for caregivers of people with dementia

Authors	Study design	Caregivers					Care-receivers		
		***N***	Mean Age (SD)	% female	Type of caregiver		Mean Age (SD)	% female	Type of dementia
Pollak & Stokes 1997 [19]	CS	25	67.3 (10.2)	24	I		80.7 (7.9)	52	AD VD UD
Akkerman & Ostwald 2004 [20]	RCT	5	–	–	I		–	–	AD
Ancoli-Israel et al. 2005 [21]	RCT	63	GLM: 67.7 (15.9) DPZ: 69.4 (11.4)	52 53	I		76.5 (7.7) 77.8 (6.2)	68 56	AD
McCurry et al. 2005 [22]	RCT	36	IG: 62.8 (15.3) CG: 63.7 (16.7)	76 68	I		77.8 (8.1) 77.6 (6.7)	41 47	AD
Lee et al. 2007 [23]	PCS	39	67.4	67	I		76.5	33	AD UD
McCurry et al. 2008 [24]	CS	44	64.6 (15.2)	67	I		78.8 (7.2)	50	AD
Rowe et al. 2008 [25]	CS	31	70.7 (7.8)	74	I		–	–	–
Beaudreau et al. 2008 [26]	CS	60	64.8 (12.5)	100	I		81.2 (7.2)	–	–
Merrilees et al. 2009 [27]	CR	1	53	100	I		58	0	FTD
Simpson & Carter 2010 [28]	Non-RCT	10	63 (14.85)	50	I		–	–	–
Higgins et al. 2010 [29]	MCS	1	73	100	I		80	0	VD
Rowe et al. 2010 [30]	RCT	49	IG: 61.52 (13.53) CG: 62.81 (10.50)	74 88	I		80 (8.58)	53	AD LBD UD
Marquez et al. 2012 [31]	CS	24	68.6 (9.1)	75	I		–	–	AD UD
Merrilees et al. 2013 [32]	CS	22	bvFTD: 59.9 (9.1) svFTD: 63 (10.8)	64	I		bvFTD: 61.5 (5.9) svFTD: 66.2 (8.9)	36	FTD
Schwartz et al. 2013 [33]	CS	126	74.16 (7.98)	71	I		–	–	AD
Merrilees et al. 2014 [34]	CS	22	bvFTD: 59.9 (9.1) svFTD: 63 (10.8)	64	I		bvFTD: 61.5 (5.9) svFTD: 66.2 (8.9)	36	FTD
von Känel et al. 2014 [35]	LS	126	74.16 (7.98)	71	I		–	–	AD
D’Aoust et al. 2015 [36]	CS	53	63.15 (12)	81	I		–	–	–
Sakurai et al. 2015 [37]	CS	20	60	80	I		–	–	–
Figueiro et al. 2015 [38]	CS	34	71.8 (12.3)	79	I		80.8 (7.9)	26	AD
McCrae et al. 2016 [39]	CS	55	62.8 (12.21)	78	I		–	–	–
Fowler et al. 2016 [40]	RCT	28	IG: 60 (12.77) CG: 67 (12.2)	27 69	I		85 (9.71) 78 (8.64)	–	–
Smagula et al. 2017 [41]	CS	57	74 (7.4)	77	I		–	–	AD UD
Peng et al. 2019 [42]	CS	43	65.40 (9.84)	93	I		77.40 (9.02)	30	AD VD UD
Gibson & Gander 2019 [43]	CS	15	72	87	I		80	–	AD VD LBD UD
Sadeghi et al. 2019 [44]	CS	8	–	75	I		–	–	–
Kajiwara et al. 2019 [45]	CS	9	65 (8.9)	44	I		83.1 (11.5)	67	AD LBD UD
Lai Kwan et al. 2019 [46]	CS	3	–	67	I		–	100	–
Sakurai & Kohno 2020 [47]	CS	10	65.05 (9.7)	60	I		83.2 (11.8)	40	–
Chang et al. 2020 [48]	CS	43	65.40 (9.84)	93	I		–	–	–
Carpenter et al. 2020 [49]	CS	14	59.4 (6.9)	100	I		–	–	AD
Song et al. 2022 [50]	CS	122	74.31 (8)	70	I		–	–	AD
Chen et al. 2022 [51]	CS	22	65.4 (8.9)	32	I		61.7 (14.2)	68	AD UD
Smagula et al. 2023 [52]	CS	56	71 (6.7)	68	–		–	–	–
de Dios-Rodriguez et al. 2023 [53]	RCT	176*	IG: 59 (–) CG: 69 (–)	76 67	I		81.5 (–) 82 (–)	69 59	–
Farina et al., 2024 [54]	CS	26	76.4 (5.9)	73	I		79.8 (5.8)	38	–
Song et al. 2024 [55]	RCT	30	67 (10.9)	93	I		82.9 (9.4)	37	AD VD LBD UD

*I* informal caregivers; *AD* Alzheimer’s disease; *VD* vascular dementia; *FTD* frontotemporal dementia; *bv* behavioral variant; *sv* semantic variant; *LBD* Lewy body dementia; *UD* dementia of unknown cause; *RCT* randomized controlled trial; *CS* cross-sectional study; *L* longitudinal study; *PCS* prospective case series; *MCS* methodological case study; *CR* case report; *IG* intervention group; *CG* control group; GLM: galantamine; DPZ: donepezil; SD: standard deviation

– not specified

^*^ study in which the numbers of caregivers (176) and care-receivers (140) were different

Except for the work of Pollak & Stokes [19], published in 1997, the included studies were published between 2004 and 2024 and had different study designs. All studies involved informal caregivers, except for one study where the type of caregivers was not specified [52], with a predominantly female population. Care-receivers in most studies (19/37) were individuals with Alzheimer’s disease or dementia of unknown cause.

### Caregiver outcome measures

**Fig. 2:**
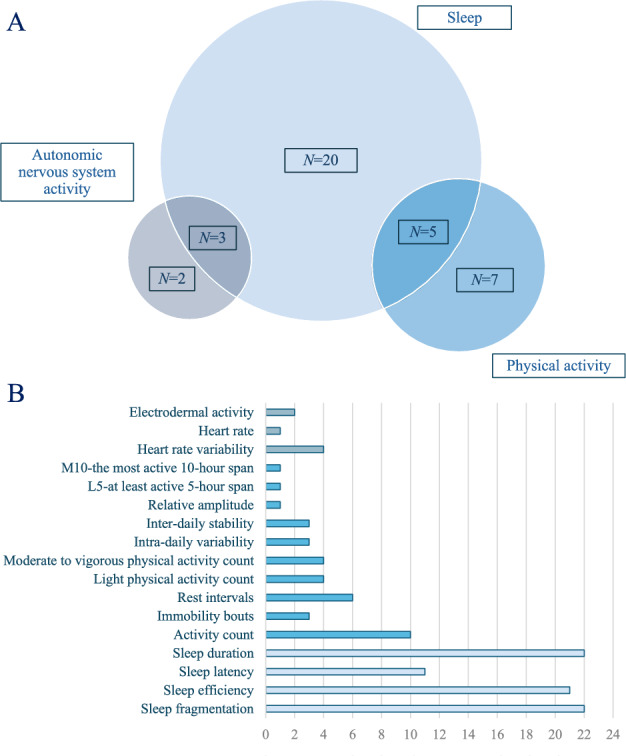
Objective outcomes (**A**) and variables (**B**) measured with wearable sensors in caregivers of patients with dementia in the selected studies

The most evaluated caregiver outcomes were sleep quality and quantity (28/37), followed by physical activity (12/37). Metrics of autonomic nervous system (ANS) activity, i.e., heart rate variability and electrodermal activity, were the focus of five studies. Eight studies focused on sleep combined with either physical activity evaluation (5/37) or ANS metrics (3/37). The results are summarized in Fig. 2. Further details are provided in Supplementary Table 2 (S2).

#### Sleep quality and quantity

Sleep quality and quantity were evaluated based on different outcome measures such as sleep duration (22 studies), sleep efficiency (21 studies), sleep latency (11 studies), and sleep fragmentation (22 studies). These studies had variable aims, such as comparing people with dementia with their caregivers (four studies), comparing caregivers with a non-caregiver control group (three studies), and evaluating the effectiveness of interventions (eight studies).

The results of the studies that compared caregivers with non-caregivers were controversial. Rowe et al. found that caregivers had longer sleep latency, lower sleep duration and efficiency, and greater night-to-night sleep variability than non-caregivers [25]. In contrast, other studies observed no significant differences between caregivers and non-caregivers [37, 50]. However, according to self-reported questionnaires on sleep quality and quantity, caregivers presented significantly worse sleep than non-caregivers [37, 50].

The results of the studies assessing people with dementia and their caregivers highlighted poor sleep quality of both members of the caregiving dyad [24, 27, 29, 34]. It is noteworthy to mention the finding of McCurry 2008 et al., wherein the adverse sleep outcome measures in one member of the caregiving dyad were not directly correlated with the evidence of sleep disruption in the other member [24].

Eight studies examined the efficacy of various interventions to enhance the sleep quality of caregivers. According to Akkerman & Ostwald, a cognitive-behavioral intervention for caregivers improved sleep duration and fragmentation [20]. Figueiro et al. showed that lighting improved caregivers’ sleep duration and efficiency in winter compared to summer [38]. McCurry et al. found that a comprehensive sleep education program significantly improved sleep duration and fragmentation of caregivers [22]. No other intervention enhanced the sleep outcome measures in the other selected studies [21, 28, 30, 40, 55].

Two studies assessed the impact of respite care, which is temporary institutional care provided to people with dementia to alleviate the burden of their usual caregivers, on the sleep quality of caregivers [23, 47]. Lee et al. conducted a sleep analysis before, during, and after 2 weeks of institutional respite care, evaluating sleep differences between caregivers who shared or did not share the bedroom with the care receiver. The sleep analysis revealed that non-sharer caregivers demonstrated the greatest improvement in sleep duration during respite, while sharer caregivers did not increase sleep duration [23]. In a study by Sakurai & Kohno, there was no significant improvement in sleep outcome measures after one night of “short-stay services” respite care [47].

#### Physical activity

The caregivers’ physical activity and rest-activity rhythm were evaluated in 12 studies based on different outcome measures, including activity counts, rest interval duration, number and duration of immobility bouts, intra-day variability, and inter-day stability of the rest-activity rhythm. The main objectives were to compare either people with dementia with caregivers or caregivers with non-caregivers and to evaluate the effectiveness of different interventions.

Similar rest-activity patterns [19] and similar levels of physical activity [31] were reported between caregivers and non-caregivers. According to Marquez et al., caregivers and non-caregivers only differed in the duration of light-intensity activity in the early morning, with caregivers appearing to be less active [31].

The included studies showed that caregivers were more active than people with dementia and that there was a positive association of physical activity within the dyad [19, 32, 49, 54].

Two studies investigated the effectiveness of different interventions to improve physical activity and the rest-activity rhythm of caregivers but found no significant results [38, 53].

Lastly, Merrilees et al. conducted a 2-year monitoring of a caregiver, revealing a progressive decrease in activity counts, an increase in the duration of the rest interval, an increase in the number and duration of inactivity bouts, and a decrease in inter-day stability [27].

#### Autonomic nervous system activity

Five studies focused on evaluating the autonomic nervous system in caregivers, assessing heart rate, heart rate variability, electrodermal activity, and skin temperature.

Kajiwara et al. showed a significant increase in heart rate after vs. before caregiving [45]. Lai Kwan et al. evaluated an intelligent assistive technology for significant-moment detection (i.e., moments of significant interpersonal connection) based on heart rate variability, electrodermal activity, and skin temperature, in contrast to conventional subjective reports [46]. The intelligent assistive technology identified distinct and personal characteristics of physiological reactivity in each participant, demonstrating that personalized algorithms can identify meaningful moments experienced by both dyad members with good agreement with subjective reports [46].

According to the research conducted by Sakurai et al., caregivers demonstrated a significantly higher level of sympathetic nervous system activity during sleep in comparison to non-caregivers despite the absence of any objective distinctions in the sleep measures [37]. However, this conclusion was based on higher values of the LF/HF index of heart rate variability, a disputed indirect index of cardiac sympathetic activity [56]. In a subsequent study by the same author, the LF/HF index was significantly lower in caregivers on respite days than on caregiving days but only during the first half of the sleep period [47].

### Subjective outcome measures

A total of 15 studies assessed the potential correlation between objective measurements obtained through wearable sensors and subjective outcome measures related to the health and well-being of caregivers and people with dementia, including depression, anxiety, quality of life, and burden of care. Depressive symptoms among caregivers were associated with lower sleep efficiency [26, 42], greater sleep fragmentation [41, 52], and longer sleep latency [42]. In contrast, no significant correlation was found between objective sleep measures and depression in the study by D’Aoust et al. (D’Aoust et al., 2015). Moreover, longer sleep latency was associated with a more significant burden of care and fatigue perceived by caregivers [42, 48], and lower sleep efficiency was associated with a more significant burden of care and poorer self-rated health status [26, 42]. No significant correlation was reported between caregivers’ objective sleep measures and positive or negative affect measures [35, 39]. Lastly, Chen et al. related caregivers’ anxiety with the physical activity of the dyad, reporting that a lower in-phase physical activity linkage between caregivers and non-caregivers was associated with greater caregiver anxiety [51].

#### Feasibility and acceptability of wearable sensors

Only two studies have provided information regarding the acceptability of wearable sensors, and they have yielded a satisfactory acceptance rate among caregivers [28, 29]. It is worth mentioning that these studies are pretty old and that wearable sensors’ form factor and usability have improved significantly since then.

### Methodological considerations on wearable sensor systems

**Fig. 3:**
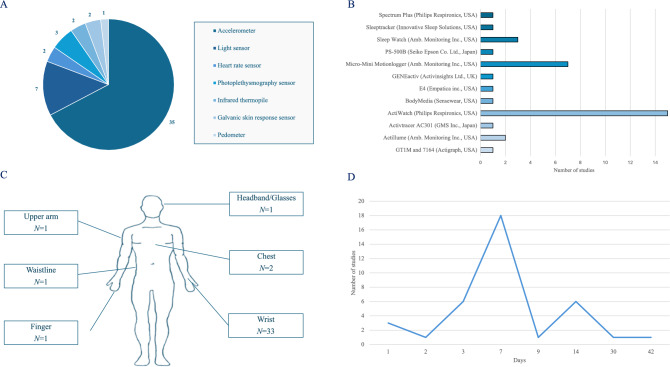
Information on the wearable sensors used in the selected studies on caregivers of people with dementia, i.e. the type of sensors (**A**), the actigraphy devices used in the studies (**B**), the sensor placement locations (**C**), and the length of the recordings (**D**)

Figure 3 shows information on the wearable sensors used in the selected studies, i.e., the type of sensors, the actigraphy devices used, the sensor placement locations, and the length of the recordings. Further details are provided in the Supplementary Table 3 (S3).

A single device was used in 33 studies, with the wrist being the most common sensor placement location (33 studies), in particular at the non-dominant wrist (16 studies). Caregivers were monitored for one or more weeks in 27 studies, particularly for one week in 18 studies, 9 days in one study, two weeks in six studies, and more than 2 weeks in two studies. Among the most used actigraphy devices, the Actiwatch (Philips Respironics, Murrysville, PA, USA) was selected in 15 studies, while the Micro-Mini Motionlogger watch (Ambulatory Monitoring Inc., Ardsley, NY, USA) was selected in 7 studies.

## Discussion

### What is already known

The application of wearable sensors to caregivers of people with dementia has never been directly assessed with a critical review of the published literature. Only two reviews in the scientific literature marginally addressed this topic [12, 13]. Gao et al. published a systematic review with a meta-analysis of the sleep quantity and quality of caregivers of people with dementia [12]. However, most included studies assessed sleep using subjective measures, such as self-reported questionnaires, with only nine studies focusing on outcomes derived from wearable sensors. More recently, Mattos et al. evaluated the relationship between care burden and sleep quantity and quality in caregivers of people with dementia with a scoping review, but only four of the included studies evaluated outcomes measured with wearable sensors [13].

### What this study adds

This scoping review provides the first comprehensive map of the published evidence on the use of wearable sensors in caregivers of people with dementia. We identified and selected 37 reports, which largely exceeds the number of reports on wearable sensors discussed in previous reviews that marginally addressed this topic [12, 13]. Sleep measures were the most frequently evaluated outcomes (28/37). Eight reports assessed the effectiveness of different interventions targeting both caregivers and care receivers and found no improvement in caregivers’ sleep; three compared the sleep of caregivers of people with dementia with controls, with conflicting results; five focused on depressive symptoms; and four associated these symptoms with shorter sleep duration and greater sleep fragmentation and sleep latency. Physical activity was evaluated in 12 reports, again with conflicting results. A single device was used in 33 reports and sensors were most commonly placed at the wrist (33/37). Most studies monitored caregivers for one or more weeks (27/37). No studies were conducted on formal caregivers.

### Strengths and limitations

The strengths of our study include a systematic approach, the use of database queries with broad inclusion criteria, the search conducted on multiple databases, and the research team, which includes authors from different backgrounds. A limitation of our review is represented by the exclusion of grey literature, such as study protocols. Further work should summarize information from study protocols that explored the use of wearable sensors in dementia care, searching databases of various funders, such as the European Horizon and the Active Assisted Living (AAL) programs. Another limitation of our review is that we did not address the integration of wearable devices with other health monitoring technologies, such as smart home sensors and telemedicine platforms, in monitoring caregivers of people with dementia. This promising field warrants future work. Finally, no quality appraisal of the selected studies was performed, although this is not a specific requirement of scoping reviews.

### Conclusion and research agenda

For caregivers of people with dementia, the potential applications of wearable sensors remain incompletely explored, with significant gaps in our understanding due to limitations of the available evidence. It is still unclear whether information obtained with wearable sensors may help predict and prevent burnout and adverse health effects due to the care burden of caregivers of people with dementia and ameliorate the disease trajectory of their care receivers.

More studies are needed with specific study designs (caregivers vs. non-caregivers and pre- vs. post-interventions) and including formal caregivers. Specific research on the potential of multi-wearable sensor systems, including > 1 sensor sites and ANS metrics, is warranted in caregivers of people with dementia. In addition, even though the limited available evidence on the feasibility and acceptability of wearable sensors in this specific population is promising, it needs to be confirmed, increasing sample diversity in terms of formal/informal caregiving, age, gender, and sensor location.

Although wearable sensors are promising tools, their use in monitoring caregivers of people with dementia still presents several challenges. First, as caregivers are often older adults who may not be familiar with technology, targeted strategies to improve the adoption of wearable devices are needed, as adherence to continuous monitoring can be inconsistent. Providing education and support to help caregivers effectively use and benefit from these technologies could be helpful to this aim. Second, given that wearable sensors manage personal health data, it is critical to properly manage privacy issues and ensure the security of devices and data storage systems to mitigate the risks of data breaches when sharing personal health data with healthcare providers or third-party applications. Third, while short-term studies indicate positive effects, limited long-term research validates the sustained benefits of wearables for dementia caregivers. Enhancing adherence to wearable devices would allow investigation of any sustained benefits of wearable device use for caregivers of people with dementia.

## Supplementary Information

Below is the link to the electronic supplementary material.

Supplementary file1 (DOCX 23 KB)

Supplementary file2 (DOCX 37 KB)

Supplementary file3 (DOCX 30 KB)

## Data Availability

All data relevant to the study are included in the article or uploaded as online supplemental information.
