# Supplementary material for: Wearable sensors for monitoring caregivers of people with dementia: a scoping review
Source: Eur Geriatr Med. 2024 Dec 3;16(2):473–83. doi: 10.1007/s41999-024-01113-8 (PMC12014814; doi:10.1007/s41999-024-01113-8)
Supplement: Supplementary file 1 — Supplementary file1 (DOCX 23 KB) [file 41999_2024_1113_MOESM1_ESM.docx]

Supplementary Table 1. Search Strategy

| **Pubmed**: 1 AND 2 AND 3 | |
| --- | --- |
| 1 | (((("wearable electronic devices"[MeSH Terms]) OR ("photoplethysmography"[MeSH Terms])) OR ("accelerometry"[MeSH Terms])) OR (photoplethysmograph* OR ppg OR ("smart glasses"OR smartglasses OR smartwatch* OR "smart watch*" OR "smart ring*" OR "smart shirt*" OR "smart glove*" OR "smart patch*" OR "smart band*"OR smartphone* OR wrist*))) OR ("wearable sens*" OR "wearable device*" OR "wearable technolog*" OR "portable sensor*" OR "portable device*" OR "portable technolog*" OR "body sensor network" OR acceleromet* OR "inertial sensor*"OR "inertial measurement unit*" OR imu OR imus OR actigraph* OR gyroscop* OR magnetomet* OR baromet* OR pedometer* OR "motion sensor*" OR "movement sensor*"OR "activity sensor*" OR "fitness tracker*") |
| 2 | caregiver* OR carer* OR family OR health personnel |
| 3 | dementia OR Alzheimer |

| **PsycINFO**: 1 AND 2 AND 3 | |
| --- | --- |
| 1 | "smart glasses" OR smartglasses OR smartwatch* OR "smart watch*" OR "smart ring*" OR "smart shirt*" OR "smart glove*" OR "smart patch*" OR "smart band*" OR smartphone* OR wrist* OR photoplethysmograph* OR ppg OR "wearable electronic devices" OR "wearable sens*" OR "wearable device*" OR "wearable technolog*" OR "portable sensor*" OR "portable device*" OR "portable technolog*" OR "body sensor network" OR acceleromet* OR "inertial sensor*" OR "inertial measurement unit*" OR imu OR imus OR actigraph* OR gyroscop* OR magnetomet* OR baromet* OR pedometer* OR "motion sensor*" OR "movement sensor*" OR "activity sensor*" OR "fitness tracker*" |
| 2 | caregiver* OR carer* OR “family member*” OR “health personnel” |
| 3 | dementia OR Alzheimer |

| **WOS**: 1 AND 2 AND 3 | |
| --- | --- |
| 1 | ("smart glasses" OR smartglasses OR smartwatch* OR "smart watch*" OR "smart ring*" OR "smart shirt*" OR "smart glove*" OR "smart patch*" OR "smart band*" OR smartphone* OR wrist* OR photoplethysmograph* OR ppg OR "wearable electronic devices" OR "wearable sens*" OR "wearable device*" OR "wearable technolog*" OR "portable sensor*" OR "portable device*" OR "portable technolog*" OR "body sensor network" OR acceleromet* OR "inertial sensor*" OR "inertial measurement unit*" OR imu OR imus OR actigraph* OR gyroscop* OR magnetomet* OR baromet* OR pedometer* OR "motion sensor*" OR "movement sensor*" OR "activity sensor*" OR "fitness tracker*") |
| 2 | (caregiver* OR carer* OR “family member*” OR “health personnel”) |
| 3 | (dementia OR Alzheimer) |

| **IEEE**: 1 AND 2 AND 3 | |
| --- | --- |
| 1 | ("All Metadata": actigraphy OR barometer OR pedometer OR motion sensor OR movement sensor OR activity sensor OR fitness tracker OR wearable OR portable OR "body sensor network" OR accelerometer OR gyroscope OR magnetometer OR photoplethysmography OR smart OR inertial sensor OR inertial measurement unit OR imu OR imus) |
| 2 | [("All Metadata":alzheimer OR dementia )](https://ieeexplore-ieee-org.ezproxy.unibo.it/search/searchresult.jsp?contentType=all&filter=-ContentType+EQ+%22Newsletters%22&matchBoolean=true&searchField=Search_All&queryText=(Search_All:alzheimer+OR+dementia+)&history=no) |
| 3 | ("All Metadata": caregiver OR carer OR health personnel) |

| **Scopus** |
| --- |
| ( TITLE-ABS-KEY ( caregiver* OR carer* OR “family member*” OR “health personnel” ) ) AND ( TITLE-ABS-KEY ( dementia OR alzheimer ) ) AND ( ( TITLE-ABS-KEY ( "wearable electronic devices" OR "wearable sens*" OR "wearable device*" OR "wearable technolog*" OR "portable sensor*" OR "portable device*" OR "portable technolog*" OR "body sensor network" OR acceleromet* OR "inertial sensor*" OR "inertial measurement unit*" OR imu OR imus OR actigraph* OR gyroscop* OR magnetomet* OR baromet* OR pedometer* OR "motion sensor*" OR "movement sensor*" OR "activity sensor*" OR "fitness tracker*" ) ) OR ( TITLE-ABS-KEY ( photoplethysmograph* OR ppg ) ) OR ( TITLE-ABS-KEY ( "smart glasses" OR smartglasses OR smartwatch* OR "smart watch*" OR "smart ring*" OR "smart shirt*" OR "smart glove*" OR "smart patch*" OR "smart band*" OR smartphone* OR wrist* ) ) ) AND ( LIMIT-TO ( SRCTYPE , "p" ) OR LIMIT-TO ( SRCTYPE , "j" ) ) |
