# Supplementary material for: Wearable sensors for monitoring caregivers of people with dementia: a scoping review
Source: Eur Geriatr Med. 2024 Dec 3;16(2):473–83. doi: 10.1007/s41999-024-01113-8 (PMC12014814; doi:10.1007/s41999-024-01113-8)
Supplement: Supplementary file 2 — Supplementary file2 (DOCX 37 KB) [file 41999_2024_1113_MOESM2_ESM.docx]

Supplementary Table 2. Objective and subjective outcomes measured in the selected studies.

| **Authors** | **Study objectives** | **Caregiver** | | | | |  | **People with dementia** | |
| --- | --- | --- | --- | --- | --- | --- | --- | --- | --- |
|  |  | **Objective outcomes measured with Wearable sensors** | |  | **Subjective outcomes** | |  | **Subjective outcomes** | |
|  |  | **Outcomes** | **Variables** |  | **Outcomes** | **Tools** |  | **Outcomes** | **Tools** |
| Pollak & Stokes (1997) | Measuring subjective sleep quality and motor activity patterns in older people with and without dementia and their caregivers. | PA | AC, IB |  | – | – |  | – | – |
| Akkerman & Ostwald (2004) | Evaluating the efficacy of cognitive–behavioral intervention for caregivers. | Sleep | SF |  | – | – |  | – | – |
| Ancoli–Israel et al. (2005) | Evaluating the efficacy of Galantamine or Donepezil on sleep in people with dementia and their caregivers. | Sleep | SF, SE, SD |  | – | – |  | – | – |
| McCurry et al. (2005) | Evaluating whether a sleep education program could improve sleep in people with dementia and their caregivers. | Sleep | SF, SE, SD |  | – | – |  | Cognitive functioning.  Depression | MMSE  CSDD |
| Lee et al. (2007) | Evaluating the sleep–wake patterns of people with dementia and their caregivers before, during, and after 2–week periods of institutional respite care. | Sleep | SF, SE, SL, SD |  | – | – |  | – | – |
| McCurry et al. (2008) | Measuring the day–to–day variation in sleep characteristics and the concordance between nighttime sleep of people with dementia and their family caregivers. | Sleep | SF, SE, SD |  | Depression  Health status  Other | CES–D  SF–36  DMSS |  | Cognitive functioning  Depression | MMSE  CSDD |
| Rowe et al. (2008) | Measuring the sleep patterns in caregivers who provide care during the night and compare those patterns to non-caregiver adults. | Sleep | SF, SE, SL, SD |  | – | – |  | – | – |
| Beaudreau et al. (2008) | Measuring whether distress and disease burden were associated with objective measures of sleep disturbance in caregivers. | Sleep | SF, SE, SL, SD |  | Depression  Health status | BDI  1 item from MOS short-form general health survey |  | Cognitive functioning  Nighttime behavior | MMSE  1 item from RMBPC |
| Merrilees et al. (2009) | Measuring the longitudinal changes in rest–activity patterns in a single male with dementia and the effects of this movement on his caregiver. | Sleep  PA | SF, SE, SD  AC, IB, RA, IV, IS, L5, M10 |  | – | – |  | – | – |
| Simpson & Carter (2010) | Evaluating the feasibility of a sleep intervention for caregivers of people with dementia. | Sleep | SF, SE, SL, SD |  | – | – |  | – | – |
| Higgins et al. (2010) | Measuring light exposure and rest–activity patterns in a single male with dementia and his caregiver. | Sleep  PA | SF, SE, SL, SD  IV, IS |  | – | – |  | – | – |
| Rowe et al. (2010) | Evaluating if a nighttime home monitoring system, designed to track the movements of people with dementia, would relieve worry and improve sleep in their caregivers. | Sleep | SF, SE, SD |  | – | – |  | – | – |
| Marquez et al. (2012) | Measuring and compare subjectively and objectively measured PA, PA preferences, psychosocial determinants of PA, and mental health indicators between older non-exercising caregivers and non-caregivers. | PA | AC, LPA, MVPA |  | – | – |  | – | – |
| Merrilees et al. (2013) | Measuring daytime activity in people with dementia and their caregivers. | PA | AC, IB |  | – | – |  | Apathy | NPI |
| Schwartz et al. (2013) | Measuring whether sleep duration and efficiency were associated with prevalent diabetes, hypertension, and dyslipidemia among caregivers. | Sleep | SE, SD |  | – | – |  | – | – |
| Merrilees et al. (2014) | Measuring sleep in people with dementia and their caregivers. | Sleep | SE, SD |  | – | – |  | – | – |
| von Känel et al. (2014) | Measuring the relationship between positive affect and sleep in caregivers. | Sleep | SF, SE, SD |  | Affect | PANAS |  | BPSD | RMBPC |
| D'Aoust et al. (2015) | In caregiver of people with dementia, measuring a set of factors that are modifiable and associated with high levels of depression in other population and determining whether these factors are predictive of depression after controlling for non–modifiable, demographic, and clinical factors. | Sleep | SF, SE, SL, SD |  | Depression  Burden  Affect | CES–D  ZBI  PANAS |  | – | – |
| Sakurai et al. (2015) | Measuring autonomic nervous system activity and quality of sleep in caregivers and non-caregivers. | Sleep  ANS | SF, SE, SL, SD  HRV |  | Stress | Visual analog scale |  | BPSD | DBDS |
| Figueiro et al. (2015) | Evaluating the effectiveness of a lighting intervention designed to increase circadian stimulation during the day for people with dementia and their caregivers. | Sleep  PA | SE, SL, SD  IV, IS |  | – | – |  | – | – |
| McCrae et al. (2016) | Measuring sleep and affect association in caregivers | Sleep | SF |  | Affect | PANAS |  | – | – |
| Fowler et al. (2016) | Evaluating the feasibility and effectiveness of an Interprofessional Virtual Healthcare Neighborhood on insomnia and sleep quality of caregivers. | Sleep | – |  | – | – |  | – | – |
| Smagula et al. (2017) | Measuring the associations of sleep with non–sleep depression symptom severity among strained caregivers who were currently without clinical depression. | Sleep | SF  SD |  | Depression | HRSD |  | – | – |
| Peng et al. (2019) | Measuring factors associated with caregivers’ sleep. | Sleep | SF, SE, SL, SD |  | Sleep  Depression  Burden  Health status | SHI  CES-D  CBI  CIRS |  | Sleep | SDI |
| Gibson & Gander (2019) | Testing the reliability of automatically scored actigraphy data as an alternative to the traditional manual method. | Sleep  PA | –  AC, RI |  | – | – |  | – | – |
| Sadeghi et al. (2019) | Testing a clinical decision support system to predict sleep quality based on trends of physiological signals in deep sleep. | Sleep  ANS | SF, SE, SD  HRV, EDA |  | – | – |  | – | – |
| Kajiwara et al. (2019) | Measuring the relationship between caregiver burden and caregiver pulse rate. | ANS | HR |  | Burden | ZBI |  | – | – |
| Lai Kwan et al. (2019) | Testing a novel intelligent assistive technology for the detection of significant-moments based on patterns of physiological signal changes in people with dementia and their caregivers. | ANS | HRV, EDA |  | – | – |  | – | – |
| Sakurai & Kohno (2020) | Evaluting of changes in caregiver sleep during short–term care | Sleep  ANS | SF, SE, SL, SD  HRV |  | – | – |  | – | – |
| Chang et al. (2020) | Measuring factors associated with fatigue among caregivers of people with dementia. | Sleep | SF, SE, SL, SD |  | Fatigue | VAS–F |  | – | – |
| Carpenter et al. (2020) | Measuring the prevalence of obesity among caregivers of people with dementia and assessing their levels of sedentary behavior and PA. | PA | AC, RI, LPA, MVPA |  | – | – |  | – | – |
| Song et al. (2022) | Measuring the discrepancy between self–reported and objective SD, considering the role of caregiving status (caregivers vs non-caregivers). | Sleep | SD |  | Depression  Affect | CES–D  PANAS |  | – | – |
| Chen et al. (2022) | Measuring how linkage in couples’ daily somatic activity (e.g., synchronized movement measured from wrist sensors) was associated with relationship quality and mental health in caregivers and people with dementia. | PA | AC, RI |  | Anxiety  Depression  Relationship quality | BAI  CES-D  MAT |  | – | – |
| Smagula et al. (2023) | Measuring how levels of PA throughout the day differed in caregivers with and without depression symptoms and testing whether such differences predicted changes in symptoms 6 months later. | Sleep  PA | SF  AC, RI |  | Depression | PHQ–9  CES–D |  | – | – |
| de Dios–Rodríguez et al. (2023) | Evaluating the effectiveness of an intervention designed to increase PA among people with dementia and their caregivers. | PA | AC, RI, LPA, MVPA |  | – | – |  | – | – |
| Farina et al. (2024) | Measuring the  relationship between PA of people with dementia and their caregivers. | PA | AC, RI, LPA, MVPA |  | – | – |  | – | – |
| Song et al. (2024) | Evaluating the effectiveness of a sleep intervention program for people with dementia and their caregivers. | Sleep | SF, SE |  | – | – |  | – | – |

**Abbreviations**: PA: physical activity; AC: activity counts; IB: immobility bouts; RI: rest interval; SF: sleep fragmentation; SE: sleep efficiency; SL: sleep latency; SD: sleep duration; LPA: light physical activity counts; MVPA: moderate to vigorous physical activity counts; HRV: heart rate variability; HR: heart rate; EDA: electrodermal activity; IS: inter-day stability; IV: inter-day variability; ANS: autonomic nervous system; BPSD: Behaviour and Psychological Symptoms of Dementia scale; NPI: Neuropsychiatric Inventory; BAI: Beck Anxiety Inventory; CES–D: Center for Epidemiological Studies Depression scale; HRSD: Hamilton Rating Scale for Depression MMSE: Mini Mental State Examination RMBPC: Revised Memory and Behavior Problem Checklist; CIRS: Cumulative Illness Rating Scale; PANAS: Positive and Negative Affect Schedule; ESS: Epworth Sleepiness Scale; VAS–F: Visual Analog Scale for Fatigue; PSQI: Pittsburgh Sleep Quality Index; CSDD: Cornell Scale for Depression in Dementia; SF–36: SF–36 Health Survey; DMSS: Dementia Management Strategies Scale; BDI: Beck Depression Inventory; MAT: Marital Adjustment Test; MOS: Medical Outcomes Study; CBI: Caregiver Burden Inventory; SDI: Sleep Disorder Inventory; SHI: Sleep Hygiene Index.

– not defined
