# Supplementary material for: Wearable sensors for monitoring caregivers of people with dementia: a scoping review
Source: Eur Geriatr Med. 2024 Dec 3;16(2):473–83. doi: 10.1007/s41999-024-01113-8 (PMC12014814; doi:10.1007/s41999-024-01113-8)
Supplement: Supplementary file 3 — Supplementary file3 (DOCX 30 KB) [file 41999_2024_1113_MOESM3_ESM.docx]

Supplementary Table 3. Information on the wearable sensors used in the selected studies.

| **Authors** | **Year** | **Type of sensors** | **Device*** | **Site** | **Duration of the recording** |
| --- | --- | --- | --- | --- | --- |
| Pollak & Stokes | 1997 | Accelerometer | Micro-Mini Motionlogger (Ambulatory Monitoring Inc., Ardsley, NY, USA) | Wrist (non-dominant) | 9 days |
| Akkerman & Ostwald | 2004 | – | – | Wrist (–) | 48 hours |
| Ancoli-Israel et al. | 2005 | Accelerometer | Actiwatch  (Mini Mitter Co, Inc; Bend, OR, USA) | Wrist (–) | 7 days**^#^** |
| McCurry et al. | 2005 | Accelerometer | Actillume  (Ambulatory Monitoring, Inc., Ard-  sley, NY, USA) | Wrist (–) | 7 days**^#^** |
| Lee et al. | 2007 | Accelerometer | Actiwatch  (Mini Mitter Co, Inc; Bend, OR, USA) | Wrist (–) | 6 weeks |
| McCurry et al. | 2008 | Accelerometer | Actillume  (Ambulatory Monitoring, Inc., Ard-  sley, NY, USA) | Wrist (–) | 7 days |
| Rowe et al. | 2008 | Accelerometer | Actiwatch  (Mini Mitter Co, Inc; Bend, OR, USA) | Wrist (non-dominant) | 7 days |
| Beaudreau et al. | 2008 | Accelerometer | Micro-Mini Motionlogger (Ambulatory Monitoring Inc., Ardsley, NY, USA) | Wrist (non-dominant) | 72 hours |
| Merrilees et al. | 2009 | Accelerometer | Actiwatch  (Mini Mitter Co, Inc; Bend, OR, USA) | Wrist (non-dominant) | 14 days**^#^** |
| Simpson & Carter | 2010 | Accelerometer | Micro-Mini Motionlogger (Ambulatory Monitoring Inc., Ardsley, NY, USA) | Wrist (dominant) | 72 hours**^#^** |
| Higgins et al. | 2010 | - Accelerometer - Light sensor | - Sleepwatch-L (Ambulatory Monitoring Inc., Ardsley, NY, USA) - Daysimeter (–) | - Wrist (–) - Attached to a headband or glasses | 7 days |
| Rowe et al. | 2010 | - Accelerometer - Light sensor | ActiWatch L  (Philips Respironics, Murrysville, PA, USA) | Wrist (–) | 7 days |
| Marquez et al. | 2012 | Accelerometer | ActiGraph GT1M and 7164 (Actigraph, Pensacola, FL, USA), | Waistline | 7 days |
| Merrilees et al. | 2013 | Accelerometer | ActiWatch  (Philips Respironics, Murrysville, PA, USA) | Wrist (non-dominant) | 7 days |
| Schwartz et al. | 2013 | Accelerometer | ActiWatch L  (Philips Respironics, Murrysville, PA, USA) | Wrist (non-dominant) | 72 hours |
| Merrilees et al. | 2014 | Accelerometer | Sleep Watch  (Ambulatory Monitoring, Inc., Ardsley, NY, USA) | Wrist (non-dominant) | 72 hours |
| von Känel et al. | 2014 | Accelerometer | ActiWatch  (Philips Respironics, Murrysville, PA, USA) | Wrist (non-dominant) | 14 days |
| D'Aoust et al. | 2015 | - Accelerometer - Light sensor | ActiWatch L  (Philips Respironics, Murrysville, PA, USA) | Wrist (–) | 7 days |
| Sakurai et al. | 2015 | - Accelerometer - Heart rate monitor | - Micro Motionlogger watch (Ambulatory Monitoring, Inc., Ardsley, NY, USA) - Activtracer AC301   (GMS Inc., Tokyo, Japan) | - Wrist (non-dominant) - – | 24 hours |
| Figueiro et al. | 2015 | - Accelerometer - Light sensor | - Motionlogger watch   (Ambulatory Monitoring, Inc., Ardsley, NY, USA)   - Daysimeter (–) | - Wrist (–) - Worn as a pendant at chest length | 7 days |
| McCrae et al. | 2016 | - Accelerometer - Light sensor | ActiWatch L  (Philips Respironics, Murrysville, PA, USA) | Wrist (non-dominant) | 7 days |
| Fowler et al. | 2016 | Accelerometer | Sleeptracker  (Innovative Sleep Solutions, Atlanta, GA, USA) | Wrist (–) | 14 days**^#^** |
| Smagula et al. | 2017 | Accelerometer | ActiWatch 2  (Philips Respironics, Murrysville, PA, USA) | Wrist (non-dominant) | 14 days |
| Peng et al. | 2019 | Accelerometer | ActiWatch 2  (Philips Respironics, Murrysville, PA, USA) | Wrist (non-dominant) | 7 days |
| Gibson & Gander | 2019 | - Accelerometer - Light sensor | ActiWatch 2  (Philips Respironics, Murrysville, PA, USA) | Wrist (non-dominant) | 7 days |
| Sadeghi et al. | 2019 | - PPG sensor - Accelerometer - Infrared Thermopile - GSR sensor | Empatica E4  (Empatica inc., Cambridge, MA, USA) | Wrist (–) | 14 days (nighttime) |
| Kajiwara et al. | 2019 | - PPG sensor, - Accelerometer | PS-500B  (Seiko Epson Co. Ltd., Nagano, Japan) | Wrist (–) | 72 hours |
| Lai Kwan et al. | 2019 | - PPG sensor - Infrared Thermopile - GSR sensor | eVu-TPS  (Thought Tech Ltd, Montreal, Canada) | Finger | 45 minutes**^#^** |
| Sakurai & Kohno | 2020 | - Accelerometer - Heart rate sensor | Micro Motionlogger watch (Ambulatory Monitoring, Inc., Ardsley, NY, USA)  WHS-1  (Union Tool Co., Tokyo, Japan) | Wrist (–)  Chest | 12 hours (nighttime) |
| Chang et al. | 2020 | Accelerometer | ActiWatch 2  (Philips Respironics, Murrysville, PA, USA) | Wrist (–) | 7 days |
| Carpenter et al. | 2020 | Accelerometer | BodyMedia  (Sensewear, Pittsburgh, PA, USA) | Upper Arm | 7 days |
| Song et al. | 2022 | Accelerometer | Sleep Watch  (Ambulatory Monitoring, Inc., Ardsley, NY, USA) | Wrist (non-dominant) | 72 hours |
| Chen et al. | 2022 | - Accelerometer - Light sensor | ActiWatch 2  (Philips Respironics, Murrysville, PA, USA) | Wrist (non-dominant) | 7 days |
| Smagula et al. | 2023 | Accelerometer | Spectrum Plus  (Philips Respironics, Murrysville, PA, USA) | Wrist (non-dominant) | 14 days |
| de Dios-Rodríguez et al. | 2023 | Pedometer | HJ-321  (Omron Healthcare Inc., Lake Forest, IL, USA) | – | 7 days |
| Farina et al. | 2024 | Accelerometer | GENEactiv  (Activinsights Ltd., Cambridgeshire, UK) | Wrist (–) | 30 days |
| Song et al. | 2024 | Accelerometer | Micro Motionlogger watch (Ambulatory Monitoring, Inc., Ardsley, NY, USA) | Wrist (–) | 7 days |

Abbreviations: PPG: Photoplethysmography; GSR: Galvanic skin response.

* some devices are multiple sensor systems; here are only the types of sensors used in the selected study.

**#** studies in which multiple separate sessions of recording have been repeated over time according to the different study protocols.

– not defined
